# The recent advances in the approach of artificial intelligence (AI) towards drug discovery

**DOI:** 10.3389/fchem.2024.1408740

**Published:** 2024-05-31

**Authors:** Mahroza Kanwal Khan, Mohsin Raza, Muhammad Shahbaz, Iftikhar Hussain, Muhammad Farooq Khan, Zhongjian Xie, Syed Shoaib Ahmad Shah, Ayesha Khan Tareen, Zoobia Bashir, Karim Khan

**Affiliations:** ^1^ College of Chemistry and Environmental Engineering, Shenzhen University, Shenzhen, China; ^2^ Additive Manufacturing Institute, Shenzhen University, Shenzhen, China; ^3^ Department of Mechanical Engineering, City University of Hong Kong, Kowloon, Hong Kong SAR, China; ^4^ A. J. Drexel Nanomaterials Institute and Department of Materials Science and Engineering, Drexel University, Philadelphia, PA, United States; ^5^ Department of Electrical Engineering, Sejong University, Seoul, Republic of Korea; ^6^ Shenzhen Children’s Hospital, Clinical Medical College of Southern University of Science and Technology, Shenzhen, China; ^7^ Department of Chemistry, School of Natural Sciences, National University of Sciences and Technology, Islamabad, Pakistan; ^8^ School of Mechanical Engineering, Dongguan University of Technology, Dongguan, China

**Keywords:** AI, drug discovery, machine learning, structure-activity relationship, artificial intelligence

## Abstract

Artificial intelligence (AI) has recently emerged as a unique developmental influence that is playing an important role in the development of medicine. The AI medium is showing the potential in unprecedented advancements in truth and efficiency. The intersection of AI has the potential to revolutionize drug discovery. However, AI also has limitations and experts should be aware of these data access and ethical issues. The use of AI techniques for drug discovery applications has increased considerably over the past few years, including combinatorial QSAR and QSPR, virtual screening, and *denovo* drug design. The purpose of this survey is to give a general overview of drug discovery based on artificial intelligence, and associated applications. We also highlighted the gaps present in the traditional method for drug designing. In addition, potential strategies and approaches to overcome current challenges are discussed to address the constraints of AI within this field. We hope that this survey plays a comprehensive role in understanding the potential of AI in drug discovery.

## Graphical Abstract

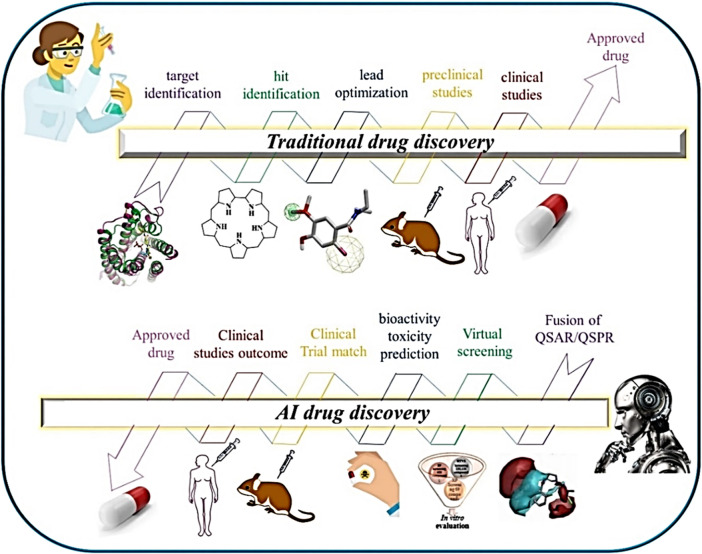

## 1 Introduction

**FIGURE 1:**
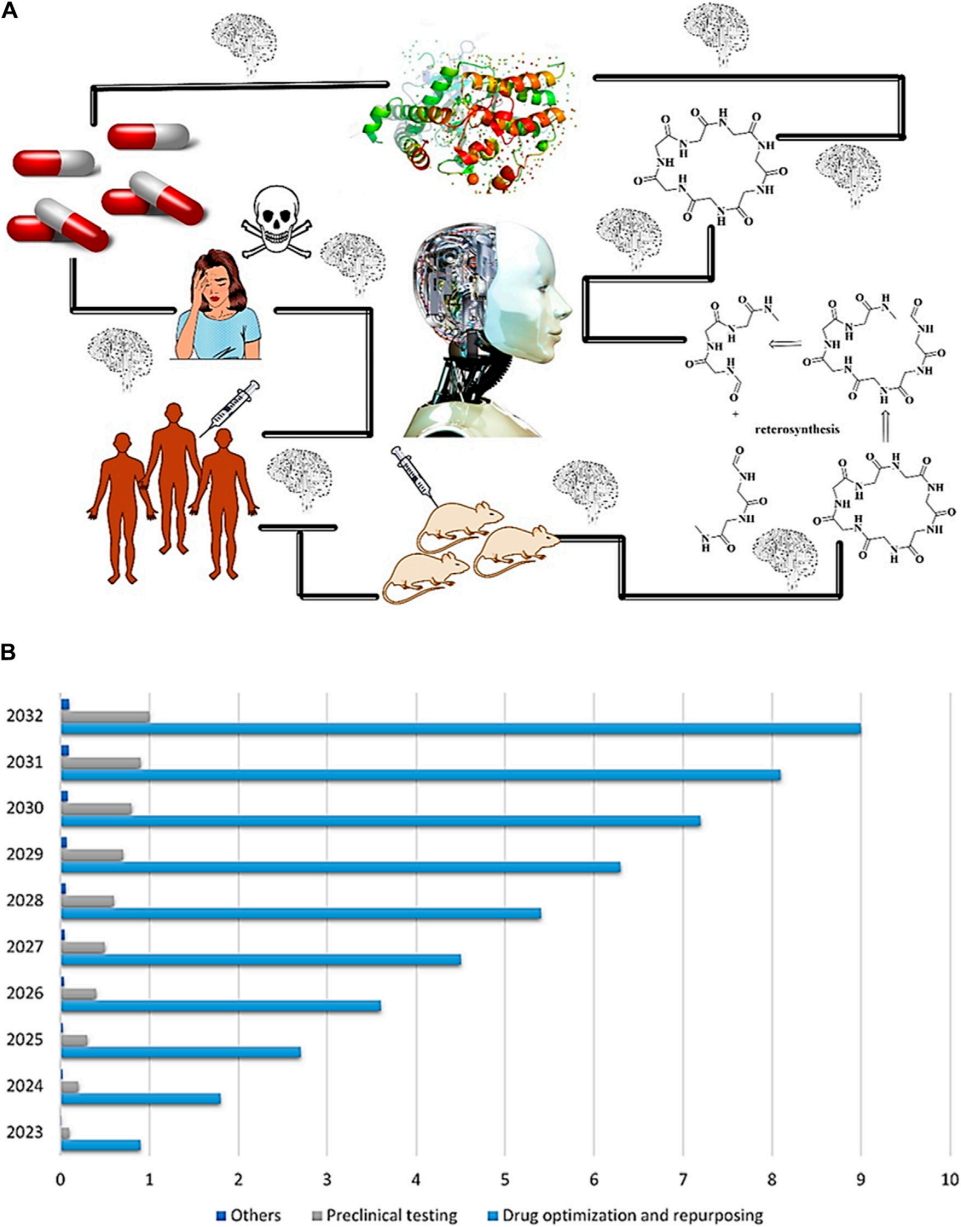
**(A)** Schematic diagram representing drug development through AI, **(B)** Significant growth in the US AI market in drug discovery is expected between 2023 and 2032.

It is estimated that 2.6 billion US dollars and over a decade of dedicated work are typically required in the field of drug discovery, which is notorious for its high costs, protracted timelines, and lack of success (Cohen et al., 2024). Several new drugs are approved, but many of these drug candidates subsequently fail. A significant precursor shift occurred in the context of drug discovery itself, enabling the rapid development of rapidly evolving artificial intelligence (AI) (Tripathi et al., 2024; Sarkar et al., 2023). Artificial intelligence has been successfully implemented into drug discovery, encompassing target protein structure identification (Hasselgren and Oprea, 2024), virtual screening (Turon et al., 2023), *de novo* drug design (Janet et al., 2023), retrosynthesis reaction prediction (Yan et al., 2023), bioactivity and toxicity prediction (Tran et al., 2023), all of which are categorized as predictive and generative processes (Figure 1A). Computer programs designed to emulate human cognitive processes constitute AI, a scientific discipline associated with intelligent machine learning. In this process, data is acquired, systems are constructed for using that data, conclusions are drawn, self-corrections are implemented, and adjustments are made where necessary (Buckner, 2023; Damiano and Stano, 2023; Prasad and Kalavakolanu, 2023; Ratten, 2024). It is generally used for the replication of cognitive tasks performed by humans through machine learning analysis. To conduct accurate analyses and provide meaningful interpretations, the technology relies on a variety of statistical models and computational intelligence (Klauschen et al., 2024). The application and integration of AI technology across diverse industries have become increasingly common in recent years (Ahmadi, 2024). Despite challenges such as shortages of pharmacists (Kilonzi et al., 2024), rising operating costs (Yaiprasert and Hidayanto, 2024), and diminished reimbursements (Pham et al., 2024), pharmacies have successfully met the rising demand for prescriptions during the past quarter-century. Pharmacy has made great strides in improving its workflow efficiency, reducing operating costs, and championing safety, accuracy, and efficiency through technology (Wilde et al., 2024). Besides giving pharmacists more time to direct their attention to a larger patient volume, automated dispensing systems improve health outcomes significantly. Intelligent automation is playing a pivotal role in improving both patient care and the pharmaceutical industry with this fusion of AI technology and pharmacy practices (Anthwal et al., 2024). The drug discovery market is expected to grow rapidly with advances in artificial intelligence technologies as well as their integration into the process as shown by Figure 1B.

## 2 Overview of artificial intelligence in drug discovery

Recent advances in artificial intelligence and machine learning have ushered in a new era of efficiency in drug discovery. By combining artificial intelligence with machine learning in drug discovery, new documents have been developed to address long-standing challenges associated with traditional drug discovery, and to accelerate the identification of promising drug candidates (Hasselgren and Oprea, 2024; Ramos et al., 2024). In computer science, artificial intelligence (AI) refers to the development of intelligent machines that can perform tasks usually requiring human intelligence. The role of machine learning in drug discovery involves analyzing vast datasets and deriving meaning from them using AI, a subset of machine learning (Kotkondawar et al., 2024).

### 2.1 Predicting drug efficacy and toxicity through machine learning (ML)

In medicinal chemistry, an important application of artificial intelligence is to predict the efficacy and toxicity of potential drug compounds. As a result, Artificial Intelligence (AI), especially Machine Learning (ML), has emerged as one of the most effective techniques for solving these problems (Alhatem et al., 2024). Analyzing large datasets allows ML algorithms to identify patterns and trends not readily evident to humans. This capability speeds up the identification of not only synthetic small molecules but also new bioactive compounds while minimizing side effects, outpacing the time constraints of traditional protocols (Thenuwara et al., 2023). For example, deep learning (DL) algorithms trained on a dataset of known drugs can predict the activity of new drugs with a high degree of success (Askr et al., 2023). The use of databases of known toxic and non-toxic compounds has enabled AI to make significant contributions to the prevention of the toxicity of potential drug compounds (Yang and Kar, 2023).

In addition to finding drug–drug interactions in patients with different diseases, AI is also essential to identifying altered or adverse reactions caused by multiple drugs being taken together for the same or different diseases (Creecy et al., 2024). The detection of drug interactions is based on AI methods that analyze patterns and trends in large datasets of known interactions. An ML algorithm, for instance, accurately predicts interactions of novel drug pairs (Atas Guvenilir and Doğan, 2023). As part of personalized medicine, AI can identify possible interactions between drugs. As a result, it is easier to develop tailor-made treatment plans based on the characteristics of individual patients, including genetic profiles and drug responses, aligned with personalized medicine, which tailor treatments based on individual characteristics (Blanco-Gonzalez et al., 2023).

### 2.2 Virtual screening: a lead identification approach

Virtual Screening (VS) serves as a potent methodology for lead identification within the domain of AI-driven drug discovery (Pun et al., 2023). By using this method, millions of compounds similar to drugs or leads are computationally screened against well-characterized proteins. Docking is used to filter ligands based on their affinities for binding (Chisholm et al., 2023; Shiota et al., 2023). These computational hits are then subjected to *in vitro* testing. Within the realm of AI drug discovery, virtual screening falls into two primary categories: ligand-based virtual screening (LBVS) (Oliveira et al., 2023) and structure-based virtual screening (SBVS) (Kumar and Acharya, 2023). LBVS entails the analysis of biological data to differentiate inactive compounds from active ones (Dragan et al., 2023). A consensus pharmacophore, similarity measure, or various descriptors are then used to identify highly active scaffolds. Conversely, SBVS requires knowledge of the 3D structure of the target protein (Rehman et al., 2023). By using computer algorithms, a target protein is docked with a large library of drug-like compounds available commercially. The docked complex is scored using a scoring function, followed by experimental validation assays (DiFrancesco et al., 2023). An important function of SBVS is scoring ligands. However, unlike ligand-based approaches, the structure-based approach does not rely on pre-existing experimental data (Stevenson et al., 2023).

## 3 Key technologies in AI–driven drug discovery

In the past decay, drug discovery was a labor-intensive process based on high-throughput screening and trial-and-error experimentation. ML and NLP techniques hold promise for improving the efficiency and effectiveness of analyzing large datasets. Improve accuracy, allowing for more precise and accurate entries through machine learning (ML) and natural language processing (NLP). (Sim et al., 2023). The recent achievements in applying deep learning to predict drug compound efficacy demonstrate AI’s transformative potential in this field. In addition, it has been proven that AI techniques are capable of projecting the criminal capabilities of an individual, showing the potential to interfere with the effectiveness of drug discovery and processing (Yang and Kar, 2023). Clearly, it is possible and research is needed on how AI can be used to create new bioactives, despite these advances and with challenges and limitations, including ethical ones. Medical advances in the future are driven in large part by artificial intelligence.

It refers to any computer or machine exhibiting responsiveness or intelligence, indicating human-like speed or intelligence, often called robotics or automation. Robotic systems are designed to perform complex repetitive tasks, while artificial intelligence is concerned with giving computers or machines the ability to think like humans (Wardat et al., 2024). As a branch of computer science, artificial intelligence (AI) aims to develop machines that can learn (Sanchez et al., 2024), organize (Nebreda et al., 2024), problem solve (Sanchez et al., 2024), sense like humans. (Akour et al., 2024), and language (Singh and Khatun, 2024) with similar success. In its current form, narrow AI, also known as weak AI, is designed for specialized tasks such as web search, face and voice recognition, and self-examination (Thangavel et al., 2024). Ultimately, the AI community wants to develop machines capable of performing all cognitive tasks better than humans, which would lead to the development of a strong or general AI.

### 3.1 A fusion of quantitative structure-activity relationship (QSAR), quantitative structure-property relationship (QSPR) and structure-based modeling

In the ever-evolving landscape of drug design, Artificial Intelligence (AI) combined with Quantitative Structure-Activity Relationship (QSAR), Quantitative Structure-Property Relationship (QSPR), and Structure-Based, has steadily gained ground in the 50 years. QSPR has proven its worth in guiding drug discovery, having proven its potential in predicting biological action and pharmacokinetic parameters (Zeng et al., 2024). As shown in Supplementary Figure S1. Traditionally reliant on simpler models, the field has progressively embraced universally applicable machine learning techniques such as support vector machines (Yin et al., 2024) and gradient boosting methods (Chellaswamy et al., 2024). Simultaneously, the resurgence of deep learning has brought forth advancements, with graph neural networks and recurrent neural networks offering automatic feature extraction capabilities (Philippe et al., 2024). This has made it possible to model complex molecular structures, including peptides (Jin and Wei, 2024) and macrocycles (Nguyen et al., 2024). Challenges, such as data scarcity and incomprehensibility, have sparked research into nature-inspired machine learning and active learning strategies. In structure-based modeling, the integration of deep learning architectures, inspired by computer vision, has revolutionized predictions for protein-ligand interactions (Xie et al., 2024). The marriage of AI with these well-established methodologies underscores a promising trajectory in drug design, with a focus on enhanced predictive accuracy and efficiency.

### 3.2 De novo drug design with artificial intelligence

The creation of novel molecular entities with desired pharmacological properties, known as *De novo* drug design, is a formidable challenge in computer-assisted drug discovery (Hasselgren and Oprea, 2024). The vast chemical space, estimated from 10^60^–10^100^ potential drug-like molecules, adds complexity. Traditional structure-based and ligand-based drug design methods, though pivotal in discovering small-molecule drug candidates, face limitations due to their reliance on specific templates derived from active sites or pharmacophores. The introduction of AI techniques has revolutionized *de novo* drug design, with models like ReLeaSE (Amilpur and Dasari, 2024), ChemVAE (Hasselgren and Oprea, 2024), Graph INVENT (Yao et al., 2023), and MolRNN (Tropsha et al., 2023) utilizing diverse molecular representations. These deep learning-based approaches accelerate the drug discovery process by exploring chemical space efficiently. Categorized as ligand-based or structure-based, these methods use rule-based or rule-free approaches (Tropsha et al., 2023). Rule-based methods involve construction rules, while rule-free approaches, often based on generative deep learning models, sample molecules from a learned latent molecular representation (Tropsha et al., 2023). These generative models, including recurrent neural networks and variation autoencoders, are praised for their efficacy in exploring chemical space. Evaluation metrics include validity, novelty, similarity to known compounds, and scaffold diversity. A promising approach combines both rule-based and rule-free methods for designing bioactive and synthesizable molecular entities (Sinha et al., 2023). While current studies predominantly focus on ligand-based approaches, there is growing interest in exploring structure-based generative design, especially for targeting orphan receptors and unexplored macromolecules.

### 3.3 Drug toxicity prediction

Prediction of drug toxicity is an essential aspect of the drug development process, with the aim of identifying and assessing the importance of potential adverse effects or adverse reactions associated with a drug in advance, when it grows further in the development pipeline. Predicting drug toxicity is important because it is critical to the safety and wellbeing of the patients who will ultimately use the drug. Predicting Drug Toxicity Traditional techniques have placed emphasis on experimental research and animal testing, which are time-consuming, expensive, and do not always accurately reflect human responses (Nasnodkar et al., 2023) and with advances in machine learning (ML), drug toxicity prediction is undergoing a paradigm shift. These techniques are based on large datasets, including chemical gradients (Nasnodkar et al., 2023), biological pathways (Guo et al., 2023), and includes information on known toxicity profiles (Dou et al., 2023). Machine learning algorithms, such as support vector machines (Khan et al., 2024), random forests (Daghighi, 2023), and neural networks (Noor et al., 2023), are trained on these data sets to learn patterns and relationships that identify potential toxicity.

The use of artificial intelligence in predicting drug toxicity offers several advantages. This enables the analysis of large data sets, allowing for a more complete understanding of the complex interactions between drugs and biological systems (Nasnodkar et al., 2023). Machine learning models can identify hidden patterns and consensual relationships that are not apparent through traditional techniques. In addition, these models can help to better and more quickly determine potential toxicities for new drug candidates, which helps in the drug development phase (Rasool et al., 2023). Yes, but challenges remain, such as the need for optimal quality, different training data, and evaluation of complex AI models. Ethical acceptance and regulatory standards also play an important role in the integration of AI-based toxicity prediction into the drug development process. Despite these challenges, there is great promise in artificial intelligence-driven drug toxicity prediction to aid the safety and success of novel pharmaceuticals (Vora et al., 2023). “Continued research and collaboration between researchers, data scientists, and regulatory agencies is essential to ensure the accuracy of the prediction of eye-driven toxicity and progress in this field.

### 3.4 Integration of AI in retrosynthesis and reaction prediction

Retrosynthesis and reaction prediction have long been crucial in organic chemistry, guiding the planning of synthetic routes. With the intersection of material science and bioscience at the bio-interface, the advent of Computer-Assisted Organic Synthesis (CAOS) (Sankaranarayanan and Jensen, 2023) has emerged as a powerful tool for synthetic planning. In recent years, the exponential growth in reaction datasets and computational power has paved the way for the development of advanced machine learning (ML) and artificial intelligence (AI) models specifically tailored for CAOS programs (Abbasi and Rahmani, 2023). These models exhibit the capability to accurately predict individual synthetic and retrosynthetic reactions, offering valuable insights for chemists in designing synthetic pathways. One notable advancement involves combining single-step predictions through the integration of proper graph search algorithms (Kassa et al., 2023). This innovative approach has allowed researchers to design CAOS programs that excel in making comprehensive synthetic pathway predictions. By leveraging the wealth of data and computational capabilities, these programs contribute to the efficiency of synthetic planning, especially in the intricate domains of material and bio-interface studies. The integration of AI and ML in CAOS not only accelerates the prediction of viable synthetic routes but also enables chemists to explore complex reaction landscapes efficiently (López, 2023). The success of these programs lies in their ability to navigate diverse chemical spaces, providing valuable guidance for designing novel compounds at the bio-interface. However, challenges persist in ensuring the reliability of predictions, addressing issues of interpretability, and refining the algorithms for diverse chemical contexts (Mittal and Ahuja, 2023). Continued collaboration between computational chemists, organic chemists, and data scientists remains essential for further advancing CAOS applications. The synergy of retrosynthesis, reaction prediction, and CAOS stands as a testament to the transformative potential of AI-driven tools in shaping the future of synthetic chemistry at the interface of materials and bioscience. Supplementary Table S1 provides a concise overview of different applications of AI in the field of drug discovery, making it easier to understand the breadth of impact.

## 4 Limitations of artificial intelligence

While artificial intelligence holds promise in drug discovery, there are significant challenges and limitations that demand careful consideration. One primary challenge is the availability of suitable data. AI-driven approaches typically rely on extensive datasets for effective training (Blanco-Gonzalez et al., 2023). However, in many instances, the accessible data may be limited, of suboptimal quality, or inconsistent, thereby compromising the accuracy and reliability of the results. Ethical considerations also present a challenge (Prem, 2023), as EI-based techniques have brought problems like fairness and biases, as discussed in the received section. For example, if the data used to train the machine learning (ML) algorithm is biased or does not properly represent the perspectives of different viewers, the unique predictions may be incorrect or invalid. Can be bent. Addressing and integrating the ethical implications of E-I is instrumental in the development of new therapeutic compounds. Different strategies can be used to meet these challenges within the scope of chemotherapy in this field. Data augmentation is a technique that involves the production of synthetic data to complement existing data sets. The amount and variety of data available for training these machine algorithms can be greatly increased, yielding and tolerating results. Other measures include the use of Explicit AI (XAI) methods, which aim to provide interpretability and transparency to the predictions of machine algorithms. Such methods contribute to addressing concerns about bias and fairness in AI-driven approaches, providing a clearer understanding of the underlying mechanisms and assumptions guiding predictions (Chen et al., 2023).

**FIGURE 2:**
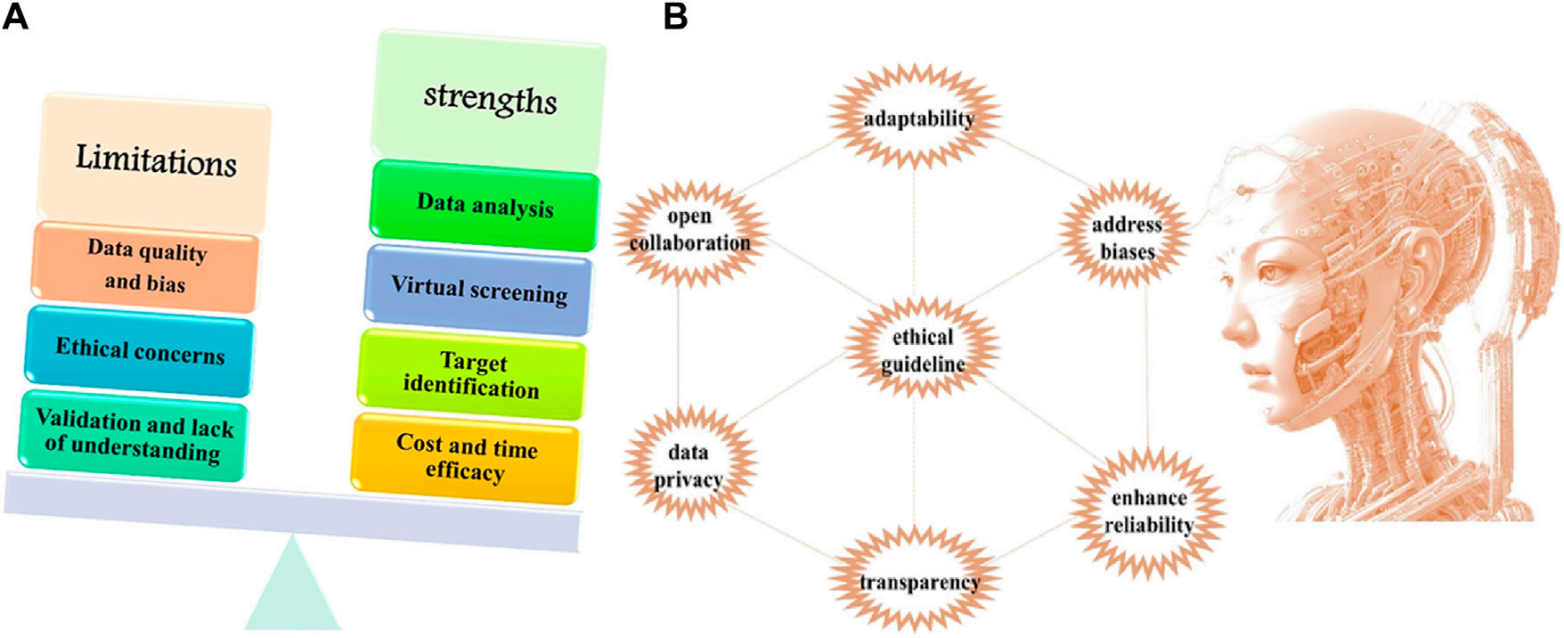
**(A)** Graphical representation of the comparison between strengths and limitations of AI, **(B)** Strategies and Approaches to Overcome Current Challenges.

Contemporary AI-based methodologies should not be viewed as a substitute for conventional experimental approaches, and they cannot replace the valuable expertise and experience contributed by human researchers (Dwivedi et al., 2023). AI is limited to offering predictions based on available data, and the subsequent validation and interpretation of results still rely on human researchers. Nevertheless, the integration of AI alongside traditional experimental methods has the potential to enhance the drug discovery process. Through the synergistic combination of AI’s predictive capabilities and the insights derived from the expertise and experience of human researchers, there exists an opportunity to optimize the drug discovery process and expedite the development of new medications (Hasselgren and Oprea, 2024; Alharbi et al., 2024; Zhang et al., 2024; Shi et al., 2022; Kang et al., 2020; Bibbò et al., 2017; Khan et al., 2020a; Iqbal et al., 2019; Khan et al., 2018a; Khan et al., 2021a; Jamil et al., 2021; Khan et al., 2020b; Tareen et al., 2021a; Khan et al., 2023; Khan et al., 2020c; Khan et al., 2021b; Tareen et al., 2022a; Khan et al., 2019a; Cao et al., 2012; Zhang et al., 2019; Hu et al., 2020; Tareen et al., 2022b; Khan et al., 2019b; Khan et al., 2021c; Khan et al., 2021d; Khan et al., 2021e; Aslam et al., 2021; Ahmad et al., 2021a; Shaheen et al., 2023; Li et al., 2023; Tang et al., 2021; Khan et al., 2019c; Khan et al., 2019d; Khatoon et al., 2020; Khan et al., 2018b; Khan et al., 2020d; Khan et al., 2018c; Khan et al., 2018d; Ahmad et al., 2021b; Duan et al., 2023; Dai et al., 2018) (Figure 2A).

## 5 Strategies and approaches to overcome current challenges

Incorporating artificial intelligence (AI) into drug discovery is a strategy of caution to overcome the current challenges. This consideration will aid in the continued development of AI in drug research. A foundational emphasis is placed on optimizing data inputs, prioritizing diverse and high-quality datasets as the bedrock for robust AI models. This addresses challenges related to data representativeness and accuracy (Figure 2B).

The establishment of ethical guidelines and governance frameworks is a critical imperative, making responsible and ethical AI use a guiding principle. This encompasses considerations such as data privacy and consent. Interdisciplinary collaboration emerges as an essential strategy, bridging the expertise of AI specialists with professionals in pharmacology, chemistry, and biology. This fosters a synergistic alliance, integrating computational capabilities with domain-specific knowledge. Transparency in AI decision-making gains significance, with the integration of Explainable AI (XAI) techniques instrumental in providing a clear understanding of AI-driven insights, particularly in the nuanced landscape of drug discovery. Adaptability is a key consideration, with the development of AI systems capable of continuous learning, ensuring sustained relevance in the dynamic field of drug discovery.

Holistic integration of computational predictions with traditional experimental methods is proposed, enhancing the reliability of drug discovery processes by capitalizing on the strengths inherent in both methodologies. Addressing biases within AI models becomes a central focus, with rigorous evaluations and mitigation strategies imperative to promote fairness and prevent disparities in drug discovery outcomes.

Engagements with regulatory bodies based on principles of quality and validation are supported to enable acceptance and regulation of AI-based tools in drug discovery. The driving force behind AI research is to promote open collaboration and data sharing that creates a culture of shared growth in the area of drug discovery.

Finally, the recommendation for investment in education and skill development programs serves to bridge the knowledge gap, ensuring a proficient workforce capable of navigating the intersection of AI and pharmaceutical sciences. In conclusion, these strategies collectively shape a comprehensive framework for overcoming existing challenges and optimizing the role of AI in advancing drug discovery methodologies (Chen et al., 2021; Sagar et al., 2021; Sagar et al., 2024).

## 6 Conclusion and summary of the potential of AI for revolutionizing drug discovery

A paradigm shift in pharmaceutical research and development is being brought about by the integration of AI into drug discovery processes. With the advent of AI, drug discovery pipelines have been significantly accelerated, offering novel solutions to longstanding challenges, such as identifying target protein structures, conducting virtual screenings, designing new drugs, predicting retrosynthesis reactions, bioactivity and toxicity. The scientific community and society overall must recognize the implications of AI-driven drug discovery moving forward. In the coming years, AI will have a significant impact on the drug development process, as it can streamline processes, reduce costs, and improve the efficiency and success rate of the identification of viable drug candidates. Furthermore, AI technologies could revolutionize patient care by improving medication management and improving healthcare delivery with the integration of AI technologies into pharmacy practices. In future, it is imperative to address several key issues. It is most important to develop new methods tailored to specific drug discovery challenges and optimize existing AI algorithms. It is also essential to integrate AI into existing drug discovery workflows seamlessly and foster collaboration among researchers, industry stakeholders, and regulatory bodies to ensure that AI is used in drug development in a responsible and ethical manner. As a result, the ongoing evolution of AI in drug discovery offers great promise for transforming the pharmaceutical sector and improving global health. It is possible to develop faster and more efficiently safer, more effective medications using AI-driven innovation and collaboration.
