# Supplementary material for: The recent advances in the approach of artificial intelligence (AI) towards drug discovery
Source: Front Chem. 2024 May 31;12:1408740. doi: 10.3389/fchem.2024.1408740 (PMC11176507; doi:10.3389/fchem.2024.1408740)
Supplement: Supplementary file 1 [file DataSheet1.docx]

**Supplementary data**

**The recent advances in the approach of artificial intelligence (AI) towards drug discovery**

**Mahroza Kanwal Khan^1^, Mohsin Raza^2,*^, Muhammad Shahbaz^2,^, Iftikhar Hussain^3,4^, Muhammad Farooq Khan^5^, Zhongjian Xie^6^, Syed Shoaib Ahmad Shah^7^, Ayesha Khan Tareen**^8^**, Zoobia Bashir^1^, & Karim Khan^2,*^**

1. College of Chemistry and Environmental Engineering, Shenzhen University, Shenzhen 518060, People’s Republic of China
2. Additive Manufacturing Institute, Shenzhen University, Shenzhen 518060, People’s Republic of China
3. Department of Mechanical Engineering, City University of Hong Kong, 83 Tat Chee Avenue, Kowloon, Hong Kong
4. A. J. Drexel Nanomaterials Institute and Department of Materials Science and Engineering, Drexel University, Philadelphia, PA 19104, USA
5. Department of Electrical Engineering, Sejong University, 209 Neungdong-ro, Gwangjin-gu, 05006, South Korea.
6. Shenzhen Children's Hospital, Clinical Medical College of Southern University of Science and Technology, Shenzhen
7. Department of Chemistry, School of Natural Sciences, National University of Sciences and Technology, Islamabad 44000, Pakistan
8. School of Mechanical Engineering, **Dongguan University of Technology**, Dongguan 523808, China

*** = Corresponding Authors: Mohsin Raza:** [mohsinraza514@yahoo.com](mailto:mohsinraza514@yahoo.com),

**Karim Khan:** [karim_khan_niazi@yahaoo.com](mailto:karim_khan_niazi@yahaoo.com)

**Table-1 Summarize applications of AI with their brief description**

| AI applications | Description |
| --- | --- |
| Target protein structure and identification | Identification of potential drug targets by analyzing the 3D structure of proteins. |
| Virtual screening | Screening large compound libraries for potential drugs using computational methods |
| Denovo drug design | Computed-based generation of new drug candidates. |
| Retrosynthesis and reaction prediction | Identifying a target compound and predicting its synthetic route. |
| Toxicity and bioactivity prediction | Analyzing the biological activity and potential toxicity of compounds based on artificial intelligence. |
| Integration into pharmacy | Improving workflow efficiency and patient care through AI-driven automation. |


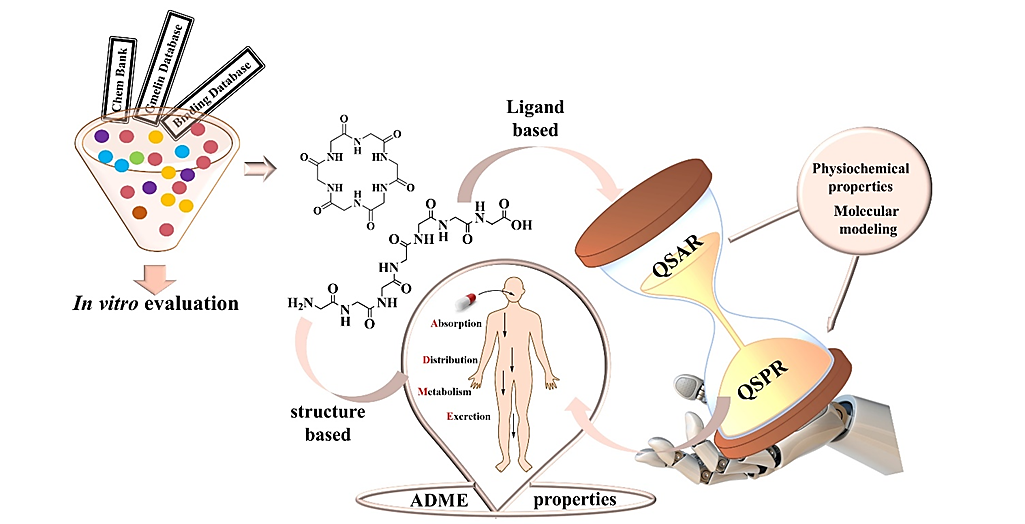


**Figure S1.** QSAR/QSPR modeling in the design of drug development.
